# Evaluating carbon-electrode dielectrophoresis under the ASSURED criteria

**DOI:** 10.3389/fmedt.2022.922737

**Published:** 2022-07-26

**Authors:** Rodrigo Martinez-Duarte, Dario Mager, Jan G. Korvink, Monsur Islam

**Affiliations:** ^1^Multiscale Manufacturing Laboratory, Mechanical Engineering Department, Clemson University, Clemson, SC, United States; ^2^Institute of Microstructure Technology, Karlsruhe Institute of Technology, Eggenstein-Leopoldshafen, Germany

**Keywords:** extreme point-of-care, carbon electrodes, dielectrophoresis, medical diagnosis, ASSURED

## Abstract

Extreme point-of-care refers to medical testing in unfavorable conditions characterized by a lack of primary resources or infrastructure. As witnessed in the recent past, considerable interest in developing devices and technologies exists for extreme point-of-care applications, for which the World Health Organization has introduced a set of encouraging and regulating guidelines. These are referred to as the ASSURED criteria, an acronym for Affordable (A), Sensitive (S), Specific (S), User friendly (U), Rapid and Robust (R), Equipment-free (E), and Delivered (D). However, the current extreme point of care devices may require an intermediate sample preparation step for performing complex biomedical analysis, including the diagnosis of rare-cell diseases and early-stage detection of sepsis. This article assesses the potential of carbon-electrode dielectrophoresis (CarbonDEP) for sample preparation competent in extreme point-of-care, following the ASSURED criteria. We first discuss the theory and utility of dielectrophoresis (DEP) and the advantages of using carbon microelectrodes for this purpose. We then critically review the literature relevant to the use of CarbonDEP for bioparticle manipulation under the scope of the ASSURED criteria. Lastly, we offer a perspective on the roadmap needed to strengthen the use of CarbonDEP in extreme point-of-care applications.

## The need for extreme point-of-care

Healthcare diagnostics is an alarming concern in rural areas worldwide, especially those in developing and least developed countries. A significant portion of the rural population worldwide deals with poor livelihood, including unhygienic domestic conditions, unavailability of safe drinking water, poor sanitation, and exposure to extreme environmental conditions, which often leads to the risk of numerous health issues (1–3). In addition, primary care in these rural areas is insufficient, along with the considerably low numbers of trained medical personnel and medical establishments. For example, there is an estimation of only one doctor in India per 1,666 habitants (4). This number gets even worse in some of the African countries. Kenya has 1 registered doctor per 6,666 of its population (5), whereas this number for Malawi, an east African country, is 1:59,900 (6). Such a poor ratio puts the healthcare professionals and the healthcare system under a tremendous burden, causing inefficiency and underperformance in primary healthcare. This has dire consequences, as an example, almost 23% of the Indian population is deprived of primary care (7, 8). Such a scenario is not exclusive to developing and least developed nations. Indigenous people in developed countries like the United States and Australia also experience limited access to primary healthcare systems due to their socio-economic conditions and racial disparity in the society (9, 10). Such lack of primary care often leads to the diagnosis of disease when it is already in an advanced state, which then requires expensive and complex treatment, seriously compromises the wellbeing of the patient, and drastically increases the economic and social burden of disease. The fact that medical treatment often comes as an out-of-pocket expense for the patient, the lack of primary care further impacts the socio-economic conditions of the population. In Nigeria, almost 96% of the total healthcare expenditure of a household pays through out-of-pocket expenses, considering the fact that 34.1% of the Nigerian population lives below the poverty line (11). In India, almost 7% of the entire population is pushed below the poverty line every year due to high health-related costs (7). Therefore, providing effective and comprehensive healthcare in resource-poor settings remains a global challenge. Extreme point-of-care (ePOC) diagnostic technologies can address some of these shortcomings by empowering the community with tools that facilitate diagnosis and thus enable effective and timely treatment.

ePOC devices enable point-of-care testing in unfavorable working environments, which are characterized by the lack of a number of basic infrastructures, including clean water, clean air and surfaces, uninterrupted power supply, stable working temperature, and trained personnel (12). The majority of the current ePOC devices are limited to lateral flow strips (LFSs), where a functionalized nitrocellulose membrane reacts with patient samples in liquid form to produce a human-readable binary result (13, 14). Low cost, easy usage, acceptable sensitivity and selectivity, and capability to use for a wide range of diseases make LFSs suitable for ePOC. However, these devices mostly focus on screening, and their use is limited in other situations mainly due to the facts that sample volume in LFS assays is mostly limited to 100 μl (15) and the inability to incorporate sample preparation. This prevents the diagnosis of conditions linked to the presence of rare cell diseases or early-stage pathogenic infections using LFS devices since such applications often require sample preparation and the analysis of several milliliters of sample volume. In such scenarios, one would benefit from ePOC devices that are able to process large sample volumes in a relatively short amount of time. In this manuscript, we postulate carbon electrode dielectrophoresis (carbonDEP) as a potential technology for ePOC technology. We assess the current progress of carbonDEP for ePOC purposes using the ASSURED criteria detailed below. We aim to identify the current limitations and propose suitable solutions to address them.

## Carbon-electrode dielectrophoresis

**Figure 1:**
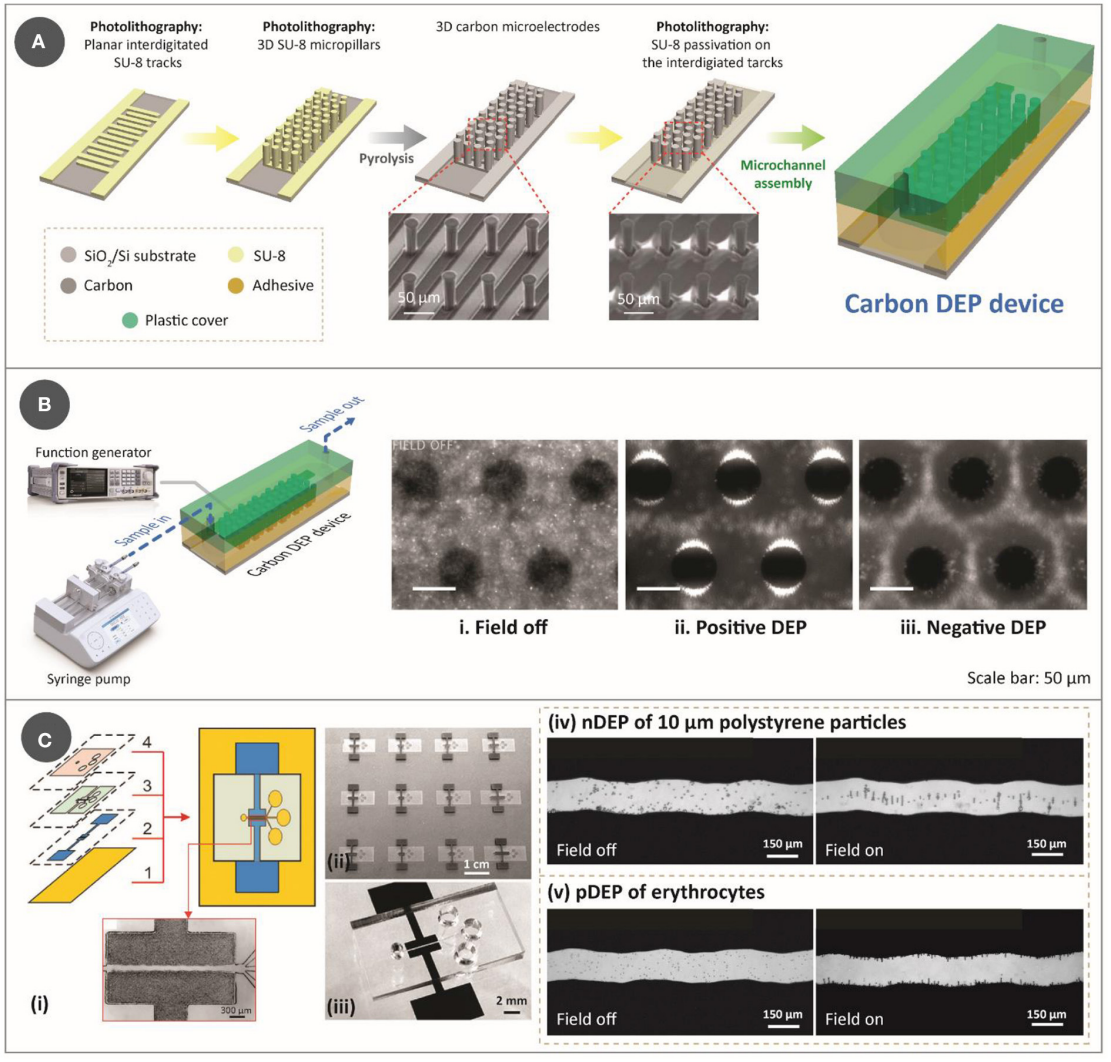
**(A)** Step-by-step process for lithography-based fabrication approach for DEP device, which features two-step photolithography of SU-8 photoresist, followed by pyrolysis and assembly with a microfluidic channel. The SEM images used to show the electrodes were reprinted with permission from Martinez-Duarte et al. (34), Copyright 2010 Royal Society of Chemistry. **(B)** Illustration of an experimental setup for a typical carbonDEP setup, consisting of a syringe pump for fluidic flow and a function generator for implementing the electric field necessary for DEP connected to the carbonDEP device. The optical images within the microfluidic channel show examples of DEP behavior. (i) Bacterial suspension around the carbon electrodes (black circles) in the absence of electric field. (ii) Positive DEP (pDEP), resulting in concentrating the bacteria at the edge of the carbon electrodes (the bright regions). (iii) Negative DEP (nDEP), repelling the bacteria from the electrodes and concentrating the cells in the empty spaces further from the electrodes. i-iii were reprinted with permission from Elitas et al. (43), Copyright 2014 Royal Society of Chemistry. **(C)** CarbonDEP using inkjet-printed carbon electrodes. (i) Layer-by-layer screen-printing fabrication of a DEP chip consisting of (1) glass substrate, (2) screen-printed carbon electrodes, (3) UV-curable paste for microchannels, and (4) top PDMS layer. (ii) Arrays of screen-printed carbonDEP devices. (iii) Individual carbonDEP device, where the black regions are screen-printed carbon. Demonstration of DEP using the screen-printed electrodes: (iv) nDEP of polystyrene particles before (left) and after (right) the electric field was applied; and (v) pDEP of erythrocytes before (left) and after (right) the electric field was applied. Reprinted with permission from Lin et al. (40), Copyright 2016 American Chemical Society.

Dielectrophoresis is a well-established technique for particle manipulation that has enabled several sample preparation steps essential for medical diagnosis, including cell separation, concentration, enrichment, and filtration (16–21). Cell sorting in DEP is mainly facilitated by the interaction between an induced electrical dipole, largely determined by the cell membrane composition and morphology, and an electric field gradient. Cells under the influence of an induced DEP force can migrate toward the maximum field gradient [known as positiveDEP, Figure 1B(ii)] or get repelled from it [negativeDEP, Figure 1B(iii)], depending on the differences between the electrical polarizability of the cell and its suspending media and the frequency of the applied electric field. Of note, the behavior of a given cell in a given suspending media can switch from positive to negativeDEP, and vice versa, depending on the frequency of the applied field. Such different behaviors are what enable the spatiotemporal manipulation of target cells that can lead to their identification and separation from a background. Over the years, several techniques have emerged to implement the electric field gradient necessary for DEP as previously reviewed by one of us (22, 23). This article particularly focuses on carbon-electrode dielectrophoresis (CarbonDEP), mainly due to the relatively inexpensive fabrication process, the robust and electrochemical inert nature of carbon electrodes, and the potential for high throughput sample processing.

### Fabrication of carbonDEP device

The typical fabrication method for carbonDEP relies on the carbonization of a patterned organic precursor to form glass-like carbon, which is an allotrope of carbon (24). Although the electrical conductivity of glass-like carbon depends on the carbonization process, usual values reported hover around 1 × 10^−4^ Ω/m (25), which is a few orders of magnitudes less than the ideal conductor but similar to indium tin oxide (26, 27). Such electrical conductivity allows implemention of DEP forces with a relatively low voltage (<20 Vpp), when the spacing between electrodes is in the order of tens of micrometers. Glass-like carbon also features excellent electrochemical stability, which has been shown to be superior to that of traditional gold or platinum electrodes. This is highly advantageous in implementing stronger DEP forces, as carbon electrodes allow the use of relatively high voltages without causing electrolysis of sample media (28). Additionally, the carbon precursors, i.e., photoresists, are significantly less expensive compared to noble metals.

The fabrication of carbonDEP devices mainly consists of three important steps: photolithography of an epoxy photoresist (typically SU-8), carbonization of the patterned photoresist, and assembly of the resulting carbon microelectrodes with a microfluidic channel (29, 30). Photolithography allows to pattern the precursor into both 2D and/or 3D shapes. 3D electrodes are advantageous over the 2D design in enhancing sample throughput. Fabrication of 3D electrodes involves two-step photolithography (Figure 1A): the first step is to pattern the photoresist into planar fingers to get the carbon connection leads, and the step includes patterning the photoresist into 3D structures over the planar base. The patterned photoresist is then carbonized, typically at 900°C in a tube furnace in an inert gas environment such as nitrogen, vacuum, forming gas, or argon. Carbonization results in near-isometric shrinkage of the electrodes (31), which varies depending on the surface area of the geometries that facilitate degassing. Of note, such shrinkage will widen the gaps of fabricated photoresist structures, and this is an important parameter to account for when designing the electrode arrays. One disadvantage of this fabrication process is the choice of substrate, as it must withstand high carbonization temperature. Current choices are limited to silicon, silicon oxide, and fused silica. After heat treatment, the fabricated carbon microelectrodes are assembled into a microfluidic network. While there are multiple ways to fabricate the microfluidic network (32), the microfluidic channels are currently fabricated out of pressure-sensitive double-sided adhesive (PSA) and polycarbonate. PSA sheets are patterned using xurography, and polycarbonate is drilled using machining. It should be noted that the use of xurography is not exclusive to carbonDEP, but is applicable to many microfluidic systems (33). The assembled device is then connected to a fluidic system to provide the sample flow within the microfluidic channel and to a function generator to supply the required electrical signal [Figure 1B (left)]. Examples of fluidic systems previously used for carbonDEP include syringe pumps and centrifugal microfluidics. In particular, the integration of centrifugal microfluidic with carbonDEP can replace the fluidic systems, such as tubings, microfluidic connectors, and syringe pumps (34).

Even though the lithography-based approach for carbon electrode fabrication is the current standard in carbonDEP, it imposes several limitations. One of them is the choice of substrate, as mentioned earlier. Furthermore, photolithography demands high-end equipment, working conditions, and skilled labor, which makes the fabrication process expensive, sophisticated, and complicated. A widely used technique to fabricate lower-cost carbon electrodes is the screen printing of carbon inks. In this technique, carbon allotropes, usually carbon black, are mixed with different matrices that lead to an ink/paste of optimized rheology (35–38). Using a stencil, this ink is deposited on a substrate in the desired pattern. Relatively inexpensive, screen printing provides a way to fabricate planar electrode arrays. Screen-printed carbon electrodes have already been demonstrated for DEP applications (39–41). Figure 1C shows an example of a screen-printed carbonDEP device, showing the layer-by-layer components of the device (40). Figure 1C(ii) shows a series of screen-printed carbonDEP devices, depicting the feasibility of line production of the devices due to their inexpensive and facile manner. Figure 1C(iv) and (v) further demonstrate the DEP behavior using the screen-printed electrodes using polystyrene particles and erythrocyte cells, respectively. The disadvantage of screen printing methods is that the achievable resolution is limited to tens to hundreds of micrometers, which is much lower than the lithography-based process. Such resolution limitation might restrict their use in specific applications where a small feature size of carbon electrodes will be required.

### Selected examples of CarbonDEP in sample preparation

A crucial step for diagnosis is the separation of targets from their background and their re-suspension in a media that facilitates analysis. The common practice in DEP is indeed to concentrate and purify a targeted population of particles, ranging from biomolecules to cells, from a sample solution. In a typical process, targeted particles are attracted to high electric field gradient regions, usually around the electrode surface, by implementing positiveDEP. This first step is then followed by the use of cell-free buffer to wash off loose and unwanted particles and then finally releasing the previously trapped particles by turning off the electric field so one can collect them at the end of the channel. CarbonDEP also works in the same principle and has been demonstrated for several sample preparation applications. We present a few exemplary applications below to show the versatility and usefulness of carbonDEP in sample preparation.

#### Improving the sensitivity of PCR-based protocols

Polymerase chain reaction (PCR) and real-time PCR are among the most popular methods of choice for medical diagnosis. Particularly, the currently ongoing COVID-19 pandemic has caused a huge surge in RT-PCR testing. Due to the huge demand, several methods have been investigated to increase PCR testing sensitivity. One of the main challenges is the presence of polymerase inhibitors in the sample that may interfere with PCR. Reported strategies to overcome PCR inhibition include sample-washing steps, density gradient centrifugation, gel electrophoresis, and column chromatography. However, these methods are often cumbersome, time-consuming, and expensive.

**Figure 2:**
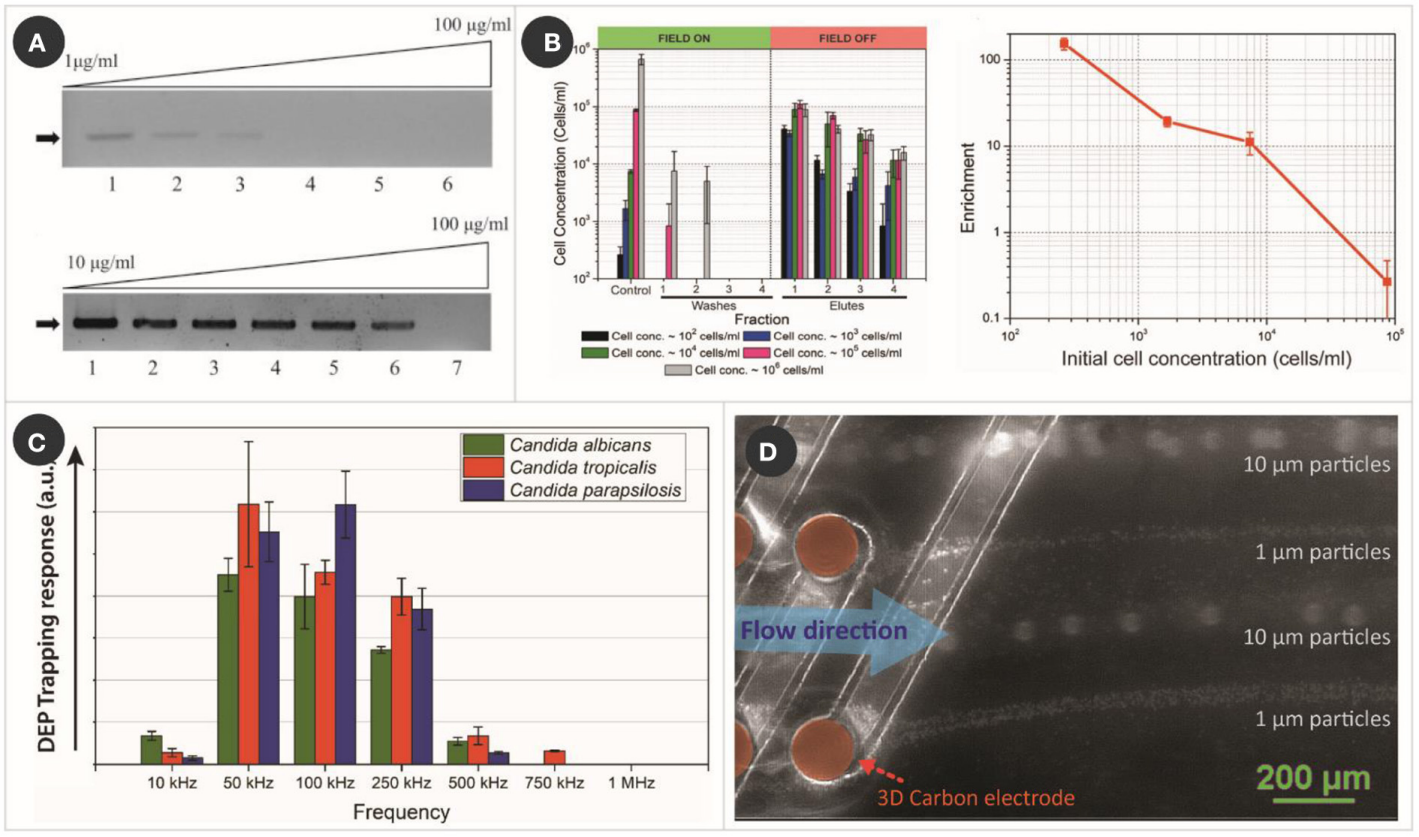
Examples of carbonDEP in sample preparation. **(A)** PCR results of humic acid spiked yeast cell sample, without (top) and with (bottom) pre-treating with 3D carbonDEP, demonstrating that 3D carbonDEP-enabled purification improved the PCR sensitivity of inhibitor-spiked samples. Reprinted with permission from Jaramillo Mdel et al. (42), Copyright 2013 Elsevier. **(B)** Experimental results (left graph) showing the enrichment of diluted samples of yeast cells with different starting concentration using 3D carbonDEP. The lowest sample concentration was 10^2^ cells/ml, and the enrichment was depicted in the first elute sample. Enrichment is plotted in the right graph, emphasizing an enrichment of 154.2 ± 23.7 for the cell concentration of 10^2^ cells/ml. Reprinted with permission from Islam et al. (47), Copyright 2016 AIP Publishing. **(C)** DEP trapping response of different strains of *Candida* using 3D carbonDEP, showing that *Candida* strains show different DEP responses based on their phenotype. This is promising in the species-specific separation of *Candida* cells for sample preparation toward the diagnosis of candida infection. Reprinted with permission from Islam et al. (50), Copyright 2020 Multidisciplinary Digital Publishing Institute. **(D)** Rapid and continuous separation of 1 and 10 μm polystyrene microparticles using 3D carbon streaming dielectrophoresis. This is promising toward implementing streaming carbonDEP for rapid and continuous cell sorting for sample preparation.

Toward increasing the sensitivity of PCR, 3D carbonDEP was demonstrated as a sample preparation module for PCR processing (42). Yeast cells from a natural sample (fermented grape must) were used as the model bioparticle, where the yeast cultures were spiked with different dosages of humic acid, the most prevalent PCR inhibitor in soils and natural surface waters, ranging from 1 to 100 μg/ml. Control samples without being processed with carbonDEP showed a sensitivity of only up to 10 μg/ml. 3D carbonDEP was implemented in a second process to successfully purify the yeast cells from samples spiked with the PCR inhibitors. The purified samples exhibited an increased sensitivity from 10 to 75 μg/ml in an assay, as shown in Figure 2A. Furthermore, carbonDEP also facilitates purifying viable cells from dead cells, reducing the possibility of false-positive in PCR.

#### Enrichment of bacteria persisters after antibiotic treatment

Bacterial persistence is a concern of clinical treatment, as it is thought to cause treatment failures, post-therapy relapses, and lengthy treatment regimens in diseases such as leprosy and tuberculosis. Furthermore, the persistence phenomenon also enhances the probability of the emergence of genetic resistance, which may cause a short lifespan of antibiotics. Therefore, there is an increasing need to understand bacterial persisters. Purifying the bacterial persister subpopulations is a viable approach to characterizing and understanding the persistence phenomenon. While fluorescence-activated cell sorting (FACS) and magnetic-activated cell sorting (MACS) are two of the most common enrichment techniques, facilitating high-throughput fractionation of cell populations, these techniques need differential labeling of the targeted cells with respective markers. However, labeling could potentially change the phenotype of the organism and affect the downstream analysis.

3D carbonDEP was demonstrated as a viable label-free tool for the isolation and purification of bacterial cells, treated with isoniazid (INH), a frontline anti-tuberculosis drug, a large enough population of bacterial cells (43). *Mycobacterium smegmatis* was used in this work since it is a model organism to investigate mechanisms of dormancy or drug-cell interactions in mycobacterial infections, such as tuberculosis. 3D carbonDEP enabled enrichment of intact *M. smegmatis* cells from a sample population of NIH-treated cells with 90% intact cells and 10% damaged cells. Enrichment of the intact cells from 90 to 99% purity was achieved, with a recovery of 3 × 10^4^ cells per assay. Such purification is advantageous in facilitating the downstream analysis of low-frequency subpopulations of cells using conventional omics techniques, such as transcriptomic and proteomic analysis.

#### Enrichment from a large sample volume of diluted cell population

Current diagnostic techniques, including PCR, mass spectroscopy, and several biosensors, require an idealized sample with a typical cell concentration of at least 10^3−4^ copies/ml (44–46). However, often pathogenic infection can start when the cell number is 1–100 copies/ml. Identification and purification of such a small pathogen population can lead to timely administration of correct antibiotics at the early stage of infection before the pathogens replicate further and cause life-threatening conditions such as sepsis. Sample preparation for such a diluted cell population is expensive, both in cost and time. There is a critical need for approaches capable of processing large sample volumes to rapidly extract and enrich the sample concentration for downstream analysis.

3D carbonDEP was demonstrated as a label-free and inexpensive alternate for processing large sample volumes and cell enrichment. Sample volume up to 4 ml featuring a cell concentration as low as 10^2^ cells/ml was processed to achieve cell enrichment (47). The cells present in such a diluted sample were trapped using positiveDEP and later re-suspend in a much smaller volume fraction (~20 μl), leading to a concentration enrichment from 10^2^ to 10^4^ cells/ml (Figure 2B). These results are highly promising in timely diagnostics of the cause of clinical sepsis, food, and environmental contamination. A maximum cell enrichment of 154.2 ± 23.7 was achieved from the diluted cell population in an assay time 6–7 h. Even though this duration can be considered long, it is considerably shorter compared to 24–48 h of currently used methods.

#### Phenotypic characterization of candida cells

*Candida* species are one of the most prevalent fungal pathogens in hospitals worldwide. Candida infection is primarily thought to be caused by *Candida albicans*. However, more than 17 other strains of *Candida* have been identified as responsible for candida infection (48). Many non-*albincas* strains do not respond to conventional anti-fungal therapy, which mostly targets *C. albicans* (49). Therefore, there is a critical need for rapid identification of the *Candida* species so that timely species-specific treatment of *Candida* infection can be initiated.

As mentioned earlier, DEP enables cell separation based on cell membrane potential and electrical field gradient interaction. Cell membrane potential differs with phenotype; therefore, phenotypical cell separation is possible using DEP. 3D carbonDEP was implemented for different strains of *Candida* to characterize the dielectrophoretic behavior of *Candida* cells (50). The three most common strains, *C. albicans, Candida tropicalis*, and *Candida parapsilosis* were investigated, which showed that the strains indeed showed different dielectrophoretic responses, as shown in Figure 2C. This study is highly promising for species-specific separation, a step close toward species-specific rapid identification.

#### Heterogenetic characterization of monocytes

Monocyte heterogeneity and its prevalence are regarded as an indicator of several human diseases, including cardiovascular diseases, chronic kidney diseases, multiple autoimmune sclerosis, ischemic brain stroke, and coronary arterial diseases (51–53). Isolated monocytes and monocyte-differentiated macrophages with preserved genetic and phenotypical properties can be used as label-free biomarkers for the timely diagnosis and treatment of various diseases. However, these immunological cells are extremely sensitive and adapt to environmental changes quickly. Therefore, cell labeling for isolating such cells might cause phenotypical and physiological alterations to the cells. Elitas et al. (54) demonstrated the use of carbonDEP as a suitable approach for label-free isolation of these cells. They first characterized the DEP response of U937 monocytes and U937 monocyte-differentiated macrophages using the 3D carbon electrode DEP device to find the crossover frequencies of the cells. Further, isolation of U937 monocytes was achieved from a cell mixture of monocytes and macrophages using carbonDEP with a separation efficiency of 70%. Elitas and Sengul further characterized the biophysical deformation of U937 monocytes and macrophages differentiated from the same monocytes under the dielectrophoretic forces (55). The monocytes exhibited a higher deformation index under DEP forces compared to macrophages from the same origin. However, the biophysical deformations were not lethal to the cells. Therefore, carbonDEP eliminates the possibility of cell damages arising from the aggressive shear forces in other flow-through separation techniques, which is advantageous for further downstream analysis.

#### Other notable work of carbonDEP

Other notable works with carbonDEP for sample preparation that are not detailed here include the implementation of rapid electrical lysis of yeast and mammalian cells (56), concentration of λ-DNA (57), and robotic transfer of captured cells from a cell suspension to a specific location (58). 3D carbonDEP was also demonstrated for streaming dielectrophoresis (Figure 2D), where cells were continuously concentrated and separated in separate streamlines by utilizing a higher sample feed rate (59, 60). This can potentially enhance the sample processing speed and throughput in sample preparation. Furthermore, carbonDEP was also integrated with a centrifugal microfluidics platform for implementing cell separation based on cell viability (34). This is significant for making the carbonDEP more compact and equipment-free and can potentially transform into a user-friendly sample-to-answer platform.

## Assessment of carbonDEP for ASSURED

In 2003, the World Health Organization Special Programme for Research and Training in Tropical Diseases (WHO/TDR) introduced a set of encouraging and regulating guidelines for the development of ePOC devices that can be used at all levels of the healthcare systems in the rural areas of developing countries for medical diagnosis and clinical management decisions (61). These criteria are known as the acronym ASSURED, which stands for Affordability (A), Sensitive (S), Specific (S), User-friendly (U), Rapid and robust (R), Equipment-free (E), and Delivered (D). In the following sections, we assess carbonDEP with reference to each of these criteria.

### A: Affordability

Affordable and cost-effective diagnosis remains the most critical aspect of ePOC, as ePOC predominantly applies to the economically poor, resource-limited rural settings. Therefore, the “affordability” criterion is thought to be the driving force in the development, approval, and uptake of new ePOC technologies. In ePOC technology, the affordability criterion is often associated with the term “low-cost,” which is assessed by the cost involved in fabrication, testing material, operating environment, and other associated costs (62). Considering the sophistication and skilled labor required for cleanroom operations and the requirement of specialized substrates, photolithography-based carbonDEP does not score well in the affordability criterion. On the other hand, screen-printed carbonDEP offers a facile and inexpensive alternative and can be suitable for the “affordability” criterion. Unlike the lithography-based approach, it does not require any special substrate for carbon electrode fabrication. Rather they can be patterned on inexpensive polymeric substrates. Recently, the laser-scribing process has also been getting significant attention as a versatile yet facile and inexpensive method for fabricating planar carbon electrodes. Laser scribing leads to direct conversation of a polymeric substrate *via* a photo-thermochemical conversion and facilitates the patterning of highly graphitic carbon material using only an inexpensive infra-red laser-engraving machine (63–65). Similar to screen-printing, it also does not require any cleanroom equipment or high-temperature furnaces and can be performed at benchtop ambient conditions, and no special substrate is needed. However, DEP performance of laser-scribed carbon DEP needs to be studied extensively, where intermediate challenges in DEP implementations need to be addressed. For example, the surface roughness and variations on the electrode edges can further lead to the non-reproducible performance of DEP devices. Furthermore, both screen printing and laser scribing methods predominantly produce 2D carbon electrodes. Toward achieving 3D carbon electrodes, micromolding and roll-to-roll patterning of carbonizable precursors are viable solutions. Another emerging technology for 3D carbon electrode fabrication is stereolithographic 3D printing, where a photosensitive epoxy resin is patterned using UV light in layer-by-layer fashion. Benchtop stereolithographic 3D printers have been already showing great promise in fabricating 3D architectures with high resolution and high printing speed. Microarchitectured 3D carbon structures have already demonstrated promising results in biomedical applications (66–68). However, its debut in DEP-based healthcare diagnostic is yet to happen. 3D printed structures need to go through oven-based carbonization process, which may be disadvantageous in terms of affordability, compared to screen printed or laser scribed electrodes. However, 3D printed carbonDEP may open novel design strategies for carbon electrodes due to the design flexibility of 3D printing, which may result surprisingly improved functionalities.

### S: Sensitive

In contrast to analytical sensitivity, or the smallest amount of a target that can be accurately measured by an assay, the diagnostic sensitivity specifies the percentage of patients who have a given condition who are identified by the assay as positive for such condition (69). The sensitivity of ePOC devices thus relates to minimizing or avoiding false negatives. This is still a major concern for current LFS devices (70). An intermediate sample preparation process can address this through isolation, purification, and enrichment of targeted bioparticles before introducing to the ePOC devices. Therefore, the sensitivity of sample preparation techniques for ePOC should be evaluated by the enabling efficiency, for example, the efficiency in isolation and purification or degree of enrichment. CarbonDEP has shown immense promise in this premise. As previously described, carbonDEP resulted in an enrichment of 154.2 ± 23.7 while processing a yeast cell sample with a concentration of 100 cells/ml (47). However, such demonstration used an optimized sample of yeast suspended in DEP-friendly media, i.e., the DEP-suitable buffer media. Processing of whole blood samples is yet to be demonstrated using carbonDEP, which would be ideal for ePOC. Furthermore, it took several hours due to a slow sample flow rate (<10 μl/min). The filtration efficiency of carbonDEP decreased significantly with a faster flow rate; in fact, it approached almost zero for a flow rate above 35 μl/min (29, 34). A crucial factor in determining the efficiency of carbonDEP is the design and dimension of the carbon electrodes, which determines the distribution of DEP force field. Smart designs of electrodes can induce strong force fields throughout the microchannel, which can avoid losing targeted cells from the starting cell sample. More extensive investigation is needed to enhance the efficiency of carbonDEP with high throughput and high sample flow rate for rapid and sensitive downstream analysis.

### S: Specific

The specificity of a diagnostic tool is an essential aspect for extreme point of care applications, as low rate of false-positive results is desired. The performance of a sensor can be improved by adequately preparing the sample. DEP has been demonstrated to be specific in general since it can detect minute differences in the DEP properties of different cells. Since such properties are given by membrane composition and size, the technique benefits from different intrinsic labels for the target. However, the differences sometimes are not as high, and other competing forces expected to be present in diagnosis devices, such as hydrodynamic forces, can mask the DEP differences. For example, carbonDEP has been used in the characterization of different *Candida* cells (50). Even though *Candida* cells of different strains feature similar cell sizes (cell diameter is within 5–8 μm), they showed slightly different DEP responses. However, there was significant overlap among their DEP responses (Figure 2C). Specificity is expected to be challenging for carbonDEP and DEP in general at sample throughput relevant for ePOC diagnostics. However, DEP can play a significant role in preparing the sample to improve the specificity of other biosensors amenable to ePOC. For example, aptamer-based biosensors have been exhibiting high promise in POC testing (71), which can be highly benefited by sample pretreatment for accurate diagnosis. A purified sample enabled by carbonDEP can minimize the possibilities of false-negative by allowing only the targeted bioparticles to be processed in the aptamer biosensor, thereby enhancing the specificity of the biosensors.

### U: User-friendly

User-friendliness refers to the capability of diagnosis with a minimal number of steps and the requirement of minimal training with no prior knowledge of diagnostic mechanisms. However, user-friendliness is a relative term of assessment. CarbonDEP, to our assessment, should not be compared with the commercial paper-based or dipstick-based diagnostic/screening test kits, as it works on a different mechanism than these commercial test kits. The current status of carbonDEP requires trained personnel for both the fabrication of the devices and the diagnosis of diseases, which may differ from the common notion of user-friendliness. However, the fabrication and sample preparation system can be potentially made significantly simpler. We have already discussed facile fabrication techniques for carbonDEP earlier. The screen-printed or laser-fabricated carbonDEP devices can be further integrated into inexpensive biosensors and detection tools, which can convert the detection results in binary “yes/no” or “on/off” signals. The sample flow system required for carbonDEP can be replaced by centrifugal force-induced flow mechanisms on a centrifugal microfluidic platform, as mentioned it earlier. Centrifugal microfluidics has already been gathering significant interest as a potential extremePOC device (72, 73). Therefore, such integration of carbonDEP and centrifugal microfluidics will not only improve the “user-friendliness index” of carbonDEP, but also make both these two technologies more relevant for extremePOC diagnosis.

### R: Rapid and robust

Extreme point-of-care devices should produce diagnostic results in a shorter period after sample collection from patients so that timely treatment can be administrated. Furthermore, the devices should function at resource-free working conditions without any need for temperature, humidity, air quality conditions, and mechanical stability during operation, storage, and transport. Toward achieving rapid cell separation, our team also demonstrated the applicability of carbonDEP in streaming dielectrophoresis, where cells were continuously concentrated and separated in separate streamlines by utilizing a higher sample feed rate (59, 60) (Figure 2D). In terms of robustness, carbonDEP technique does not need any special working conditions, as the surrounding environment has minimal influence on DEP mechanism itself. Integration of carbonDEP with a robust centrifugal microfluidics platform will further enhance the robustness index of carbonDEP.

### E: Equipment-free

Extreme POC devices, ideally, should feature a minimal number of equipment. Particularly, they should not contain any special equipment that requires extra attention, and they should be powered by solar energy or battery. Current carbonDEP platforms require an external fluidic system and a function generator to perform. As mentioned earlier, the integration of carbonDEP with a centrifugal microfluidic platform demonstrated that the fluidic system could be replaced by the inherent centrifugal force (34). However, the function generator was still connected through a slip ring. Recently our group has reported the development of an electrified-Lab-on-a-Disc (eLoaD) platform where any kind of electrical function can be supplied on-chip through wireless communication (74, 75). Furthermore, the eLoaD system features on-disk power supply modules, which can be operated through battery power as well. Integration of carbonDEP with eLoaD platforms can make carbonDEP free from external power sources, increasing the equipment-free index of carbonDEP. Further developments can be considered to make carbonDEP completely equipment free through on-chip implementation of a signal generator and power sources and enabling the fluid flow using capillary forces. Furthermore, biosensors can be integrated to carbonDEP device to enable sample-to-answer functionalities in a single device. An integrated device featuring a series of electrode arrays optimized for different functionalities can be envisioned, where first array of electrodes can be used for DEP to enrich a targeted population, the next one for cell lysis to extract their intracellular components, one more DEP arrays for enriching a targeted molecule or organelle, and a last one for detecting specific target using carbon elements functionalized with aptamers or bacteriophages (30).

### D: Delivered

Delivery of ePOC devices to the end-users should be easy and sustainable through production, procurement, transport, and distribution. The current status of carbonDEP may not favor the “delivered” criterion, mainly due to the use of silicon-based thin substrates for the production of carbon electrodes. Special care needs to be taken to procure and transport current carbonDEP devices. However, ongoing and further development of screen-printed and laser-scribed carbonDEP devices can improve this scenario significantly due to their usage of inexpensive and robust substrates. Furthermore, in the current scenario, “delivery to end-users” of carbonDEP is different from the commercial dipstick and paper-based test kits. In contrast to bedside testing, carbonDEP is currently more suitable for local healthcare centers in rural settings, where community diagnosis can be possible. Future development of equipment-free and biosensor-integrated carbonDEP devices may change this scenario and establish the integrated system as a “bedside” testing tool.

## Concluding remarks

**Figure 3:**
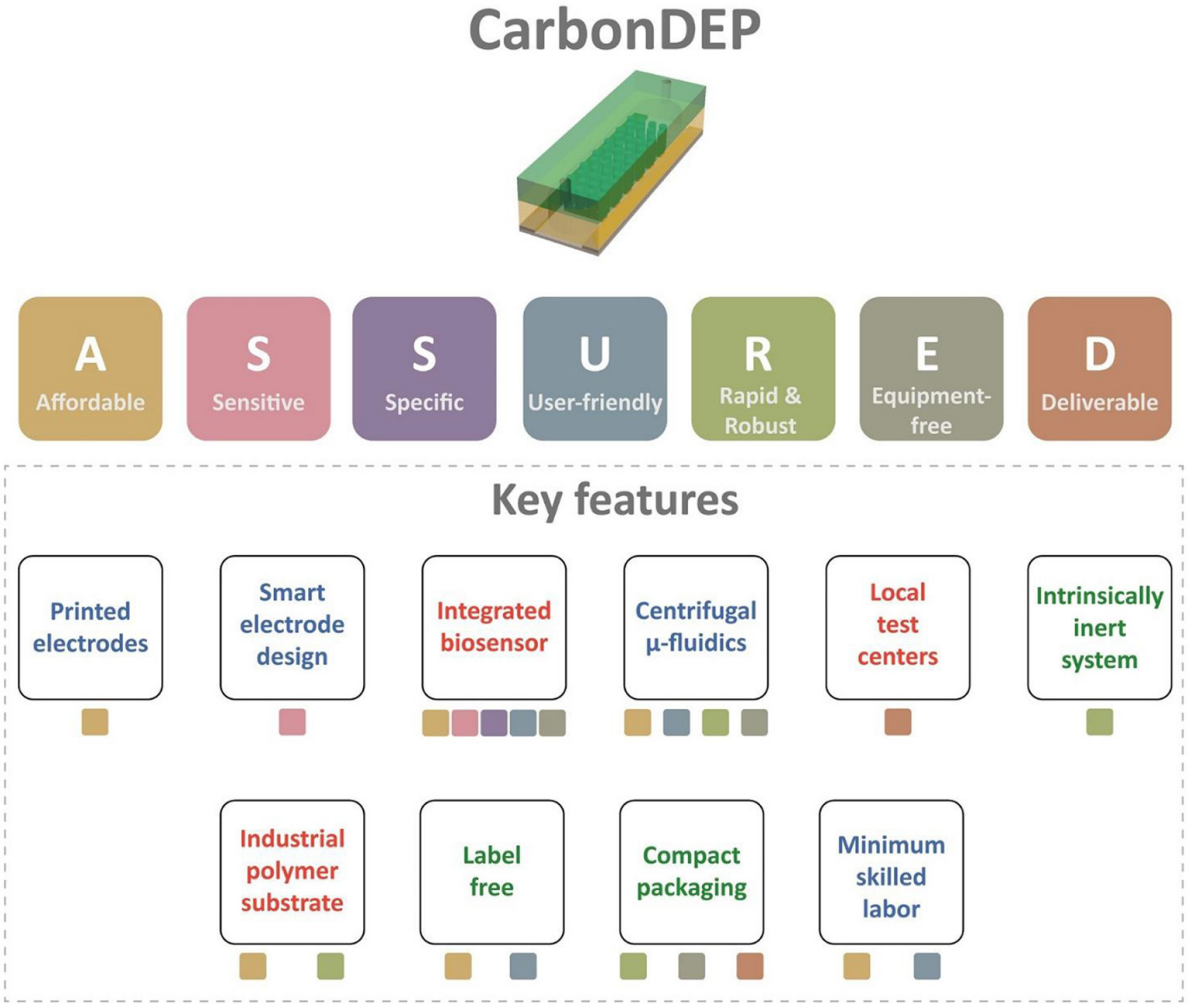
The summary of the evaluation of carbonDEP using ASSURED criteria to identify the key features suitable for ePOC applications. The color of the boxes below each feature represents the corresponding criterion from ASSURED. The features with green text represent already established protocols. The blue text signifies that initial promising results are available, but further improvement is required. The features with red texts are not proven to date, but introducing them will accelerate the usefulness of carbonDEP in ePOC applications.

This article assesses the potential of CarbonDEP for sample preparation competent in extreme point-of-care, following the ASSURED criteria. The evaluation is summarized in Figure 3. CarbonDEP has demonstrated important advances in the concentration, separation, and purification of different bio-targets, which has shown promising results in sample preparation for healthcare diagnostics. Even though carbonDEP exhibits high promise in ePOC, it requires significant further work to check each tick box under the ASSURED criteria. We have highlighted both the need and future opportunities to connect and integrate the components into functional systems. The current lithography-dominated fabrication process is expensive and requires skilled labor and sophisticated workflow. However, emerging screen printing and laser scribing can solve this by bringing a facile and inexpensive production line. Furthermore, the stereolithographic 3D printing process holds high promise in developing 3D carbon electrodes for high throughput advanced carbonDEP devices. Smart design and architecture of carbon electrodes further enhance the purification efficiency at high processing speed, which can further improve the sensitivity and specificity of downstream detection and analysis processes. Integration of carbonDEP with centrifugal microfluidics device is already a step forward toward making the platform robust and equipment-free. Further development in integrating on-chip power supply and biosensor components can lead the way toward completely equipment-free and “sample-to-answer” devices. The challenges may not be limited to the ones discussed here. There might be several unforeseen challenges during the process of development. Finding solutions to these challenges will not only further the research area of carbonDEP but may lead to other unsolved questions in system integration, biosensors, sample preparation, healthcare diagnosis, and ePOC itself.

## Data availability statement

The original contributions presented in the study are included in the article/supplementary material, further inquiries can be directed to the corresponding author/s.

## Author contributions

RM-D and MI conceived and designed the entire review and wrote the paper. All authors reviewed, edited, and approved the manuscript.

## Funding

DM, JK, and MI acknowledge support from the Deutsche Forschungsgemeinschaft (DFG, German Research Foundation) under Germany's Excellence Strategy *via* the Excellence Cluster 3D Matter Made to Order (EXC-2082/1-390761711). We acknowledge support by the KIT-Publication Fund of the Karlsruhe Institute of Technology.

## Conflict of interest

The authors declare that the research was conducted in the absence of any commercial or financial relationships that could be construed as a potential conflict of interest.

## Publisher's note

All claims expressed in this article are solely those of the authors and do not necessarily represent those of their affiliated organizations, or those of the publisher, the editors and the reviewers. Any product that may be evaluated in this article, or claim that may be made by its manufacturer, is not guaranteed or endorsed by the publisher.
